# Intermittent Fever, with Cases

**Published:** 1881-05

**Authors:** George H. Clark

**Affiliations:** Phila.


### INTERMITTENT FEVER, WITH CASES.

BY GEORGE H. CLARK, M.D., PHILA.

THAT at this day it is necessary to offer cases cured homœopathically in order to convince those who pretend to practice that exclusive system, is a cause for wonder, not to those who follow the law as laid down by Hahnemann, but to many outside of the medical profession, who have seen, and who have experienced, the benefit arising from applying that law.

To endeavor to prove the truth of a law without rigidly adhering to it, is an absurdity. To condemn without having attempted to learn whether it is true or false, is prejudice; and such condemnation is not entitled to the least respect. Many self-styled homœopaths, by their practice, (their precept, in many instances, savoring of truth), are constantly engaged in attempts —let us hope, in ignorance, or for want of knowledge—to show that the law is to be used only in some cases, while in others it is inapplicable; and notwithstanding their desire to be classed as homœopaths, they rarely try to follow, in the treatment of the sick, rules whose application is prerequisite to success.

False in one, false in all, is as true of the law governing homœopathy as of any thing, and if it can be satisfactorily shown that it is false, after an honest endeavor, it has no right to exist, and should be condemned by all right thinking, honest-minded persons. There is no affection in which rules for treatment are more explicit than in intermittent fever, and the orthodox treatment of this disease being so very easy, calling for no effort upon the part of the prescriber, that, without even trying to effect a cure homœopathically—though desiring their treatment to be called by that name—which demands painstaking labor, many are found to proclaim the inability of homœopathy to effect a cure, especially in malarial regions.

The following cases are chosen from a number, showing what may be done for such cases:

Miss W., aged twenty-four years, has resided on the eastern shore of Maryland for fifteen years, and for the past ten years she has had repeated attacks of chills and fever, which abound in that region, and the attacks have as repeatedly been suppressed with quinine. She was sent to me for treatment in the autumn of 1878. She had a tertian intermittent, the paroxysm beginning between one and two P.M. First came chill with constant thirst; then fever with continued thirst, restlessness, and mild delirium; after which, slight sweating. These symptoms had characterized each attack. After the next paroxysm had passed, Arsenicum, 60 M. (Fincke), three doses in water, at intervals of two hours, was given. To the present time (Feb. 1881) she has had no return of chills, nor any other trouble, and she has continued to reside in the same place.

A boy, aged four years, residing in the same neighborhood, had for fourteen months continued attacks of tertian intermittent. Quinine had been given until the little fellow sensibly refused to take more. In August, 1880, I learned that he had had a paroxysm every other day for seven months. The malarial and quinine cachexia was well marked, and with the chill, which began between ten and eleven A.M., there was intense thirst for large draughts of water, and during the fever which followed he complained of his head "hurting and jumping." One dose of Natrum muriaticum (30th) was given at the end of a paroxysm. He remained free from another attack until in the following October, when, the same symptoms presenting, another dose of Nat. mur. was given, and he has had no chill since, and has continued to live in the same place, and his health is constantly improving. This last case came under observation while I was visiting the section in which he resides with his parents, and on learning that many cases with symptoms of a similar character were in the neighborhood, I left a vial of Nat. mur., with directions to give one dose to any case met with; and I

learned, a few months afterward, that several cases had been cured with that remedy. Whether these last are permanent I am unable to state, but I have every reason to believe in their permanency.

There is no difficulty in treating this trouble, provided the law is adhered to. Many cases present complications that may require more than one remedy, and a longer time than was necessary in the above; but the resort to any thing but genuine treatment is unnecessary, and only likely to lead to complications that will cause much suffering, and if the patients are not familiar with homœopathy, a loss of confidence in that system. Further, there is no necessity of resorting to mysticism; fair, open dealing will make friends both for the cause and the prescriber.

Hay fever is another trouble for the cure of which resort is made to un-homœopathic treatment. Even the "regulars" acknowledge they have no help for this affection, and why he who professes a knowledge of homœopathy should resort to their non-helpful modes is to be referred to a "Philadelphia lawyer."

A lady whom I have never seen, a resident in the state of Delaware, applied by letter, in May, 1877, for treatment for hay fever. For nine years she had been subject to this distressing malady. The attack began in May and continued with more or less severity until the following September. The only marked symptoms, with those that usually characterize this affection, were excoriation of nostrils and lips from the discharge, with thirst, restlessness, while in bed, and difficulty in breathing. May 6th, 1877, four doses, dry, of Arsenicum 60 M. (Fincke) were sent, and her letter of the following week states that relief was almost instantaneous from the time of taking the first dose. Nothing more was sent until May 20th, when she wrote that the only symptoms present were continued sneezing and bland watery discharge from nostrils. Gelseminum (500th) was sent for these, and on June 4th her letter stated that she was perfectly well. This much for homœopathy. In contrast to this mode of treatment, note this: several months ago I was called to see an infant, aged nine months, whom I found in a moribund condition. On inquiry the following was elicited: "He was first taken with a cold, slight cough with discharge from nose, and Dr. Q., a homœopathic physician, treated him for two weeks but he got worse from day to day; and the medicines he gave him were so bad that the baby could not take them, and then we sent for an old-school doctor, who has treated him since that time." The child died one hour after I was called. In Dr. Q. I recognized a teacher in the "Homœopathic (?) Medical College." Is comment necessay? Can such practitioners be familiar with Hahnemann's declaration regarding such a mode of treatment? Would that they could ever have before them these words: "For eighteen years I have departed from the beaten track in medicine. It was painful to me to grope in the dark, guided only by our books in the treatment of the sick—to prescribe, according to this or that (*fanciful*) view of the nature of diseases, substances that only owed to mere opinion their place in the Materia Medica; I had conscientious scruples about treating unknown morbid states in my suffering fellow-creatures with these unknown medicines, which, being powerful substances, may if they were not *exactly* suitable (and how could the physician know whether they were suitable or not, seeing that their peculiar, special actions were not yet elucidated), easily change life into death or produce new affections and chronic ailments, which are often much more difficult to remove than the original disease. To become in this way a murderer or aggravator of the sufferings of my brethren of mankind, was to me a fearful thought—so fearful and distressing was it, that shortly after my marriage I completely abandoned practice and scarcely treated any one for fear of doing him harm, and, as you know, occupied myself solely with chemistry and literary labors.

"But children were born to me, several children, and in course of time serious diseases occurred, which, because they afflicted and endangered the lives of my children—my flesh and blood—caused my conscience to reproach me still more loudly, that I had no means on which I could rely for affording them relief.

"But whence could I obtain aid, *certain*, *positive* aid, with our doctrine of the powers of medicinal substances founded merely on vague observations, often only on fanciful conjecture, and with the infinite number of arbitrary views respecting disease in which our pathological works abound?—a labyrinth in which he only can preserve his tranquility who accepts as Gospel those assertions relative to the curative powers of medicines because they are repeated in a hundred books, and who receives, without investigation, as oracles, the arbitrary definition of diseases given in pathological works, and their pretended treatment according to hypothetical notions, as described in our therapeutical works—who does not attribute the aggravation and prolongation of the acute diseases he treats and their degeneration into chronic maladies, and the general fruitlessness of his efforts when he has to treat diseases of long standing, to the uncertainty and impotence of his art—no! he ascribes death and ill-treated disease and all, solely to the incurableness of the disease, to the disobedience of the patient, and to other insignificant circumstances, and so accommodating and obtuse is his conscience, that he satisfies himself with these excuses, though they are in many ways delusive, and can never avail before an Omniscient God; and thus he goes on treating diseases (which he sees through his systematic spectacles) with medicinal substances that are far from being without influence on life and death, but of whose powers nothing is known.

"Where shall I look for aid, *sure* aid?" sighed the disconsolate father, on hearing the moaning of his dear, inexpressibly dear, sick children. "The darkness of night and the dreariness of a desert all around me; no prospect of relief for my paternal heart!"*

* Extract from a letter to a physician on "The Great Necessity of a Regeneration of Medicine."—"Hahnemann's Lesser Writings," pp. 511, 512.

Entering into the spirit of this train of thought, when at this day, those who profess to be his followers are as much in the darkness—from their own doings—as those who opposed him when the above was written, should it excite wonder that it is still necessary to combat, as the master did, these pseudo-homœopaths?

"I do not consider any as my followers, who, in addition to leading an irreproachable, perfectly moral life, does not practice the new art in such a manner that the remedy he administers to the patient in a non-medicinal vehicle (sugar of milk or diluted alcohol) contains such a small subtle dose of the medicine, that neither the senses nor chemical analysis can detect the smallest absolutely hurtful medicinal substance, indeed not the slightest trace of any thing medicinal at all, which presupposes a minuteness of dose that must indubitably dispell all anxiety from all officers of state who have to do with medical police."*

